# Alcohol use amongst learners in rural high school in South Africa

**DOI:** 10.4102/phcfm.v7i1.755

**Published:** 2015-09-14

**Authors:** Thembisile M. Chauke, Hendry van der Heever, Muhammad E. Hoque

**Affiliations:** 1 1 Department of Public Health, University of Limpopo, Medunsa Campus, South Africa; 2 2 Graduate School of Business and Leadership, University of KwaZulu-Natal, Westville Campus, South Africa

## Abstract

**Background:**

Drinking behaviour by adolescents is a significant public health challenge nationally and internationally. Alcohol use has serious challenges that continue to deprive adolescents of their normal child growth and development. Drinking is associated with dangers that include fighting, crime, unintentional accidents, unprotected sex, violence and others.

**Aim:**

The aim of the study is to investigate drinking patterns, and factors contributing to drinking, amongst secondary school learners in South Africa.

**Method:**

The sample included 177 male (46.6%) and 206 female (53.4%) respondents in the age range from 15–23 years, selected by stratified random sampling.

**Results:**

The results indicated that 35.5% of male and 29.7% of female respondents used alcohol. Both male and female respondents consumed six or more alcohol units (binge drinking) within 30 days; on one occasion the consumption was 17.5% and 15.9% respectively. It was found that alcohol consumption increases with age, 32.2% of 15–17 year-olds and 53.2% of 18–20 year-olds consumed different types of alcohol. It was deduced that 28.9% respondents reported that one of the adults at home drank alcohol regularly, and 9.3% reported that both their parents drank alcohol daily. It was found that 27.6% of the respondents agreed that friends made them conform to drinking. The tenth and eleventh grade reported 15.2% of male and 13.9% of female respondents were aware that alcohol can be addictive.

**Conclusion:**

This study found that age, gender, parental alcohol use and peer pressure were found to be the major contributing factors to alcohol use amongst learners Prevention campaigns such as introducing the harmful effects of alcohol use amongst learners are of utmost importance in reducing alcohol use amongst learners in South Africa.

## Abstract (fr)

**La consommation d’alcool chez les élèves des collèges ruraux en Afrique du Sud.**

**Contexte:**

La consommation d’alcool chez les adolescents est un enjeu important de la santé publique sur le plan national et international. La consommation d’alcool pose des défis majeurs qui continuent à priver les adolescents d’une croissance et d’un développement normaux. L’abus d’alcool est associés à des dangers comme les bagarres, les crimes, les accidents involontaires, les rapports sexuels non protégés, la violence et autres.

**Objectif:**

Le but de cette étude est d’examiner la consommation d’alcool, et les facteurs qui contribuent à l’abus d’alcool chez les élèves des écoles secondaires en Afrique du Sud.

**Méthode:**

L’échantillon comprenait 177 hommes (46.6%) et 206 femmes (53.4%) dans la tranche d’âge de 15 à 23 ans, sélectionnés par échantillon aléatoire stratifié.

**Résultats:**

les résultats ont indiqué que 35.5% des hommes et 29.7% des femmes consommaient de l’alcool. Les hommes aussi bien que les femmes interrogés buvaient six consommations ou plus (beuveries) en 30 jours; une fois la consommation était de 17.5% et 15.9% respectivement. On a remarqué que la consommation d’alcool augmentait avec l’âge, 32.2% des 15à 17 ans et 53.2% des 18 à 20 ans consommaient différents types d’alcool. On en a déduit que 28.9% des personnes interrogées ont répondu qu’un adulte chez eux buvait régulièrement de l’alcool, et 9.3% ont dit que leurs deux parents buvaient tous les jours de l’alcool. On a trouvé que 27.6% des personnes interrogées convenaient que les amis les entrainaient à boire. Les élèves de dixième et onzième année ont répondu que 15.2% des hommes et 13.9*%* des femmes savaient que l’alcool pouvait créer une dépendance.

**Conclusion:**

Cette étude a trouvé que l’âge, le sexe, l’utilisation d’alcool par les parents et la pression des camarades étaient la cause majeure de consommation d’alcool chez les élèves. Il faudra organiser des campagnes de prévention parmi les élèves, soulignant les effets nocifs de l’alcool, si l’on veut diminuer la consommation d’alcool des élèves en Afrique du Sud.

## Introduction

Substance use, particularly alcohol, is a common source of social and health problems in almost all countries in the world, South Africa included.^1^ The World Health Organisation (WHO) (2004) states that the availability of alcohol to underage persons under different circumstances, such as alcoholic families or communities, parental permissiveness, poverty and peer pressure fuels adolescent alcohol use.^2^ Learners also seem to have drinking problems, which pose global social and public health concern.^3^ South Africa is also experiencing substantial change with the onset age of alcohol intake. According to studies conducted in South Africa it was reported that there is growing evidence that South African society experiences fairly widespread alcohol consumption amongst youth and adults.^4,5,6,7,8^

Alcohol has the potential to influence adolescents to engage in risky sexual behaviour such as multiple sex partners, and to become vulnerable to sexually transmitted infections, unintended pregnancy, and sexual violence.^9^ When a young person is under the influence of alcohol, undoubtedly the body and mind are not functioning well as expected by norms and the decision-making power is weakened. Researchers have singled out outstanding risk factors such as scholastic problems, risky sexual behaviour, crime and violence, accidents and injury. The findings indicated that early adolescent alcohol use and the use of other substances had significant negative effects on cognitive and effective self-management strategies.^10^

In South Africa, as in other countries, the alcohol initiation age has reduced significantly. The review about prevalence data from five national surveys, which were collected over 12 years in South Africa, supports the mode that binge drinking amongst youth aged 15-24 years increased between 1998–2005 from 29% to 31%.^11^ Recent studies reported high level of alcohol use amongst adolescents and high school learners in South Africa.^5,6,7,8,12^ Many studies have reported on alcohol use amongst high school learners in South Africa. However, no study has been conducted in the northern township area of Gauteng province of South Africa. Therefore, this study aimed to investigate alcohol use amongst secondary school learners in the northern township of Gauteng province, with reference to their socio-demographics characteristics, drinking patterns and contributing factors regarding substance use.

## Methodology

This was a quantitative cross-sectional descriptive study conducted at Makhosini Secondary School (MSS) in Soshanguve Township, outside the limits of the City of Tshwane Municipality in Gauteng Province, South Africa. MSS provides formal education to mainly Black African learners from neighbouring households. The school caters for learners of both sexes; Grades 10–12. The residents in the township in which the study was conducted reside in both formal and informal settlements and are of low socio-economic status.

A total of 792 students, who were in grades 10 and 11, was the population size of the study. Sample size for the study was calculated using the following information: from a population of 792 (learners) using an expected frequency of 50% and the worst expected frequency of 45% at 95 confidence level, the final sample size was 383. To prevent incomplete information the number was increased to 400 learners, the sample representative in grades 10 and 11 learners was increased to 225 and 175 respectively.

Stratified random sampling technique was used to select the sample. A table of random numbers was generated in this manner with no order of sequence, assigning a number to each of the population of 792 learners. Three hundred and ninety numbers were selected, using three digit numbers selected from the table of random numbers of three figures in columns of ten, commencing at any point in the table, accepting 30 numbers with three digits in each column horizontally, until the sample size was reached. If a number was encountered more than once, the number was skipped.

The self-administered questionnaire was developed based on previous research.^13^ The questionnaire consisted of socio-demographic factors such as age, gender, grade, and religion; the drinking patterns amongst learners; factors contributing to the use of alcohol. All the questions were closed ended. The questionnaire was written in English because it is the language of teaching and learning in the school.

Data collection day was arranged with the school head master after obtaining permission to conduct the study from the Education Department, the school governing body and the principal. Data collection was executed on the same day in a school hall, which can accommodate more than 390 learners, with a sitting arrangement that simulated a classroom situation. Confidentiality was preserved; no staff member was present or involved during the administration of the questionnaire. The researcher monitored data collection and learners were asked to complete the questionnaire and drop it inside a box next to the door. This box had a small opening on top that could prevent anyone from tampering with the contents. It was anticipated that each learner would complete the question in 20 to 30 minutes.

The data collection tool was pre-tested in a different secondary school from the school in the main study. Ten learners composed of five boys and five girls in the same grade of study were asked to volunteer in the pre-test survey after obtaining permission from the school principal. Data collection occurred between November and December 2011.

Ethical approval for the study was obtained from the ethics committee of the University of Limpopo (Medunsa Campus). Also, permission to conduct research was approved by the Gauteng Education Department, the principal and the school governing body. Learners were asked to participate in the study after the purpose of the study was explained to them and were informed that participating in the study was voluntary and that they were free to withdraw from participating at any time. All the students who were younger than 18 years were given the consent form to give to their parents for approval. Informed consent was obtained from the participants who were over 18 years of age. The parents of the respondents who were under 18 years of age provided their signatures or (thumb prints as per their wish), thereby assuring them that confidentiality would be adhered to at all times.

Data were cleaned, coded and captured and then analysed through SPSS18 (Statistical Package for Social Science). Descriptive statistics were used to analyse socio-demographic information, including age, gender and grade level. The chi-square test was carried out to find the association between categorical variables. *P*-values < 0.05 were considered statistically significant.

## Result

In the study 390 grade 10 and 11 learners took part in the study, but 383 respondents completed the questionnaire correctly. Table 1 summarises the learner's socio-demographic information. Results showed that the average age of the participants was 17.93 years with a standard deviation of 2.75 years. More than half (53.4%) were female, and 77.3% were in grade 10. Regarding religion, the majority (77.3%) were Christian followed by African Traditional (9.9%). When asked who they were currently living with, fewer than half (42.8%) of the learners responded that they were living with both parents during the study.

**TABLE 1:** Socio-demographics characteristics of the respondents.

Variables	Frequency	Percentage
**Age group**		
15–17 years	147	38.4
18–20 years	225	58.7
21–23 years	11	2.9
Mean age (SD)	17.93 (2.75) years	
**Gender**		
Male	177	46.6
Female	206	53.4
**Grade**		
Grade 10	217	56.7
Grade 11	166	43.3
**Religion**		
Christianity	296	77.3
Islam	14	3.7
African Traditional	38	9.9
Hindu	1	0.3
Other	34	8.9
**Currently living with**		
Both Parents	164	42.8
Single Parent	122	31.9
Guardian	30	8.9
Mother and step-father	34	7.8
Child-headed family	3	0.8
Grandparents	30	7.8

**FIGURE 1:**
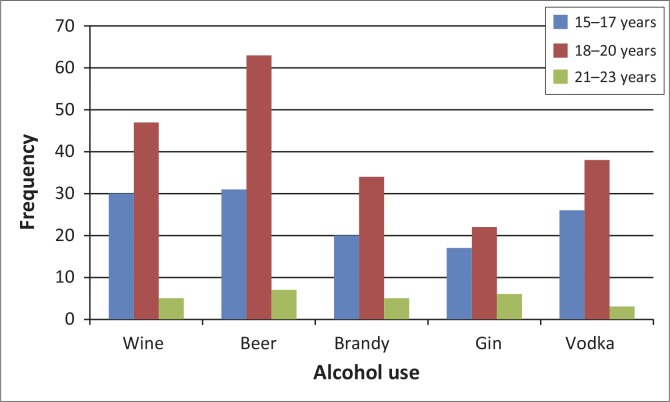
Types of alcohol used with regards to learners’ age (*n* = 354).

**FIGURE 2:**
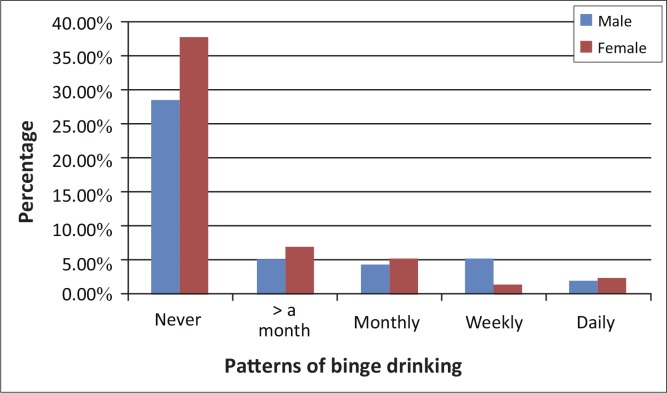
Patterns of binge drinking (six or more in one occasion) amongst the learners by gender.

It was found that 35.5% male and 29.7% female students drank alcohol in their lifetime. Results indicated that all the different age groups of learners used many different kinds of alcohol (Figure 1). With regards to binge drinking, 21.2% [95% CI: 12.7%–25.0%] reported binge drinking. Daily binge drinking was reported by nine male and eight female learners (Figure 2).

Table 2 summarises information about the effect of alcohol amongst the respondents according to gender. Thirty-nine (10.0%) of both gender reported that they did not attend school because of alcohol use. More male students (6.7%) were absent, failed to do homework (11.0%), and were unable to study for a test after alcohol consumption compared to female students (3.3%, 7.6%, and 6.3% respectively) (*p* < 0.05). It was also found that more females were embarrassed (20.3%) than male students (12.3%) because of drinking alcohol (*p* < 0.05).

**TABLE 2:** Effects of alcohol use amongst the learners.

Response	Male	Female	Total	*p-value*
**Absenting self from school because of alcohol use**				
Yes	26 (6.7%)	13 (3.3%)	39 (10.0%)	0.010
No	151 (39.4%)	193 (50.3%)	344 (89.8%)		
**Embarrassment caused by drinking**				
Yes	47 (12.3%)	78 (20.3%)	125 (32.6%)	0.02
No	130 (33.9%)	128 (33.4%)	258 (67.4%)		
**Failure to do homework after alcohol consumption**				
Yes	42 (11%)	29 (7.6%)	71 (18.5%)	0.015
No	135 (35.2%)	177 (46.2%)	312 (81.5%)		
**Unable to study for a test after alcohol use**				
Yes	37 (9.7%)	24 (6.3%)	61 (15.9%)	0.014
No	140 (36.6%)	182 (48.6%)	322 (84.1%)		

**FIGURE 3:**
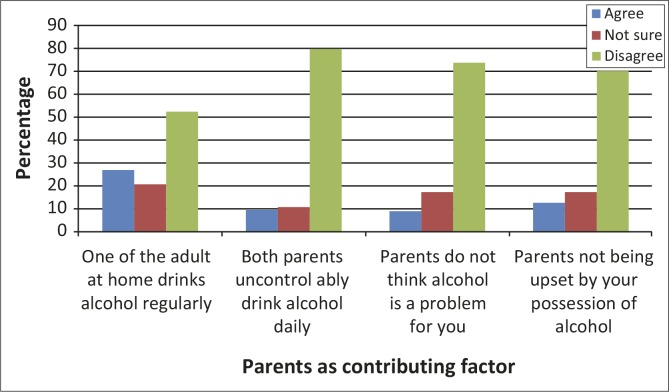
Parents as contributing factor to learners’ alcohol use (*n* = 383).

**FIGURE 4:**
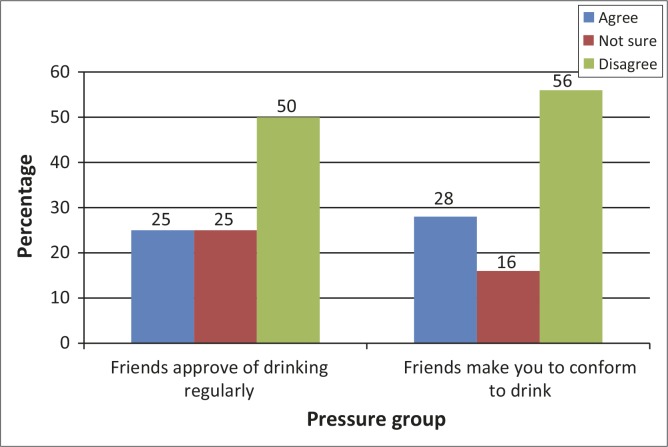
Alcohol consumption due to peer pressure group (*n* = 383).

Figure 3 shows parental behaviours as a factor contributing to alcohol use by learners. Results highlighted that more than a quarter (26.9%) of the learners reported that there was an adult that drank alcohol regularly at home and 9.3% indicated that both parents were uncontrollably drinking alcohol daily. Most respondents (73.8%) indicated that parents were unhappy about them using alcohol. With regards to peer pressure, 27.6% respondents reported that friends required them to conform to drinking, whilst 56.1% disagreed with this statement (Figure 4).

## Discussion

The present study investigated school learners’ behaviour regarding alcohol use. Alcohol use involved drinking beer, wine or spirits. In this study alcohol use is defined as the amount (quantity) consumed, patterns of drinking and how often they drank various types of alcohol. The study found risky behaviour and that it impacted on their education. Sadly, the findings of the study suggest that education and other life factors are seriously jeopardised by the pressure of alcohol use amongst learners at MSS. Young and de Klerk indicated that learners (adolescents) are faced with alcohol-related harms associated with social, health and educational problems.^14^ The study results found that learners use alcohol following various patterns, from experimenting to binge drinking.

The study found that different types of alcohol (beer, wine, brandy, gin whisky and vodka) were consumed by learners across the spectrum. The study found that beer was the most consumed liquor, followed by wine and spirit. WHO indicated that wine, beer and spirit are mostly consumed in sub-Saharan Africa, whilst the type of alcohol consumed depends on geographical differences and the type of people practicing the behaviour.^15^ A study conducted amongst adolescents in 35 European countries reported 90% of school learners used alcohol in their lifetime and 19% had engaged in illicit drug use.^16^ Studies from the USA also reported different types of substance use by the adolescents.^17,18^ The South African national Youth Risk Behaviour Survey (YRBS) reported lifetime prevalence rates for alcohol use as 50%, 13% for cannabis use and 12% for inhalants or prescription drug use.^5^ These meant that the socio-economic factors do determine the type of alcohol consumed in the region.

Binge drinking is known to be hazardous drinking where the patterns of drinking increase the chances of alcohol-related risks. It involves rapid and excessive drinking over a short period of time.^14^ The study found that both male and female respondents consumed six or more alcohol units within 30 days; on one occasion this was 17.5% and 15.9% respectively. Binge drinking has become problematic for teenagers and young people; previous studies have showed its growth in the country. There was an increase from 29% of current drinkers in 1998 to 31% in 2005 in the age group 15–24 years.^11^ The study indicated that there was less gender difference in binge drinking. This could be because of availability, accessibility, socio-economic and environmental factors. The same study added that binge drinking is a national challenge; this was proven prevalent in 24% of men in the Western Cape, followed by the North-West with 20.0%, Gauteng 16.0% and the Free State with 15.0%.^11^ This could be demonstrating how adults transfer the binge drinking behaviour to their children and society. Research conducted by the NSDUH on alcohol use and delinquent behaviour amongst adolescents aged 12–17 years found that 41.3% were involved in serious fighting at school, 22.4% had attacked someone with the intention of inflicting serious harm, and 12.4% were found carrying a handgun in the previous year. These have a negative impact on the public health.^19^

The present study illustrated that 28.9% respondents reported that one adult at home drank alcohol regularly, and 9.3% reported that both their parents drank alcohol daily. The findings indicated that parents, as role models for their children, influence their teenagers. It highlighted that parents who use alcohol have great influence on their adolescents drinking behaviour.^10^ Adolescents who are exposed to such environments at home are likely to model it and consider it to be acceptable. Such parents spend less or no quantity or quality time with their children to give support and guidance. Researchers argued that parents, who spend most of their time drinking, are linked to those adolescents who use alcohol or drugs.^10^

It was also found that 8.8% parents thought that alcohol use was not a problem for their children, whereas 12.5% respondents reported that their parents were not upset by their being in possession of alcohol. In a study it was reported that parents, who are users of alcohol, may predetermine the future alcohol involvement of their child.^3^ Parenting is a critical process during the developmental stages of a child. Most parents set boundaries clearly, for example telling their toddlers not to put things in their mouths. These set limits are necessary for a child's safety. All this changes when the child starts to talk; parents try to reason or use rewards to help their child to set and reach goals. Researchers have highlighted that adolescents that spend more time with friends than parents have greater opportunity for alcohol consumption. Parents with lower monitoring skills have a history of alcohol use.^10,20^

Some of the adolescents are involved in consumption because parents are not aware of their activities due to their lack of communication with their parents. Effective parenting is formed by the child-parent relationship. Parents’ motivation, norms, beliefs, values and goals are imperative to modify behaviour of their adolescents. More importantly, parents must be monitors and managers of adolescents’ behaviour.^20^ Parents need to discuss the critical values they want to impart, such as hard work, goal setting, showing respect, spending time with elders, and being a responsible citizen.

It was evident from the study that 25.0% of the respondents enjoyed their friends’ approval by drinking regularly; 27.6% agreed that friends made them conform to drinking. Teenagers want to be like their friends and they want to know that their friends accept them as part of the group. Adolescents pressurize each other to be homogeneous peer groups and their behaviour is sometimes outrageous. South African society is also said to be very tolerant towards alcohol and other substances. Adolescents are encouraged by some people and their peers to accept drinking, tell jokes about drinking, and wear T-shirts with slogans that promote drinking and smoking.^21^

Researchers also explain the social factors that lead adolescents to alcohol use. Curiosity and experimentation forms part of this as some see their peers becoming intoxicated with alcohol and other drugs. They also associate alcohol intake with graduating to adulthood. They also consider alcohol consumption to be normal within their peer or cultural groups.^19,21^

The population in this study comprised high school students from one school. This has implications for the generalisability of the findings. Since this is a cross-sectional study, causal inferences cannot be made from the results reported. The self-reported data are subject to bias but anonymity might have reduced this bias.

## Conclusion

This study found that a large number of learners at MSS used alcohol and engaged in binge drinking. This may contribute to risk behaviours and disruptions amongst others or the community. This study found that age, gender, parental alcohol use and peer pressure were the major contributing factors to alcohol use amongst learners Alcohol use was also found to have a negative influence on school work (e.g., absenteeism, low performance, truancy and delinquency). Prevention campaigns, such as introducing the harmful effects of alcohol use amongst learners, are of utmost importance in reducing alcohol use amongst learners in South Africa. In future, explorative research needs to be conducted to investigate why high school learners engage in risky behaviours.
